# Comparison of chemiluminiscence versus lateral flow assay for the detection of *Helicobacter pylori* antigen in human fecal samples

**DOI:** 10.1007/s10096-023-04624-7

**Published:** 2023-05-27

**Authors:** Carlos Rescalvo-Casas, Marcos Hernando-Gozalo, Laura Seijas Pereda, Carlos García Bertolín, Felipe Pérez-García, Juan Cuadros-González, Ramón Pérez-Tanoira

**Affiliations:** 1 Universidad de Alcalá, Facultad de Medicina, Departamento de Biomedicina y Biotecnología, Madrid, Spain; 2 Departamento de Microbiología Clínica, Hospital Universitario Príncipe de Asturias, Madrid, Spain; 3 Universidad de Alcalá, Facultad de Farmacia, Departamento de Química Orgánica y Química Inorgánica, Madrid, Spain; 4 CIBERINFEC, ISCIII - CIBER de Enfermedades Infecciosas, Instituto de Salud Carlos III, Madrid, Spain

**Keywords:** *Helycobacter pylori*, Antigenic test, Sensitivity, Specificity

## Abstract

*Helicobacter pylori* is a Gram-negative bacterium that causes chronic gastric inflammation, which can lead to gastric neoplasia. Therefore, early diagnosis of *H. pylori* infection is crucial for effective treatment and prevention of complications. The aim of this study was to compare the sensitivity and specificity of the *STANDARD™ F H. pylori Ag FIA stool antigen test* (SD Biosensor) with the *LIAISON® Meridian H. pylori SA* for the diagnosis of *H. pylori* infection. A total of 133 stool samples from patients with suspected *H. pylori* infection were compared using the *STANDARD™ F H. pylori Ag FIA stool antigen test* (SD Biosensor), based on lateral flow assay, with the *LIAISON® Meridian H. pylori SA*. Of the 45 positive samples with LIAISON, 44 were also positive while 1 was negative in the STANDARD™ antigen test. However, this discrepant sample showed a chemiluminescence index of 1.18, very close to the cut-off point of 1. On the other hand, of 88 negative samples obtained with LIAISON, 83 were negative and 5 were positive in the STANDARD™ antigen test. Moreover, *STANDARD™ F H. pylori Ag FIA* assay has shown a sensitivity of 97.8% (95% CI: 88.2-99.9), a specificity of 94.3% (95% CI: 87.2-98.1), a PPV of 83.9% (95% CI: 68.9-92.4) and a NPV of 99.3% ((95% CI: 95.3-99.9). In conclusion, the *STANDARD™ F H. pylori Ag FIA* (SD Biosensor) on the *STANDARD™ F2400* analyser is a highly sensitive, specific and suitable assay for the detection of *H. pylori* in stool samples.

## Introduction

*Helicobacter pylori* (*H. pylori*) is a Gram-negative, microaerophilic demanding, spirophilic, highly motile bacillus that colonizes the human gastric epithelium [1]. It is transmitted mainly by the fecal-oral and possibly by the oral-oral route [2]. Despite the decreasing prevalence of *H. pylori* infection, this bacterium still infects 30-50% of the general population in developed countries [3]. In addition, the development of *H. pylori* infection is related to the host conditions, the socioeconomic factors and the sanitary conditions. Consequently, the prevalence varies according to the geographical area and the level of development of different countries [1].

This microorganism can cause inflammation of the gastric mucosa, which in some cases can lead to complications such as dyspepsia, peptic ulcer, gastric malignancies and extra-gastric diseases [3]. Likewise, the World Health Organization detected 812,000 new cases of gastric malignancy attributable to *H. pylori* in 2018, being the third leading cause of cancer death [4].

The *H. pylori* detection techniques include both invasive (endoscopy, gastric tissue biopsy culture, gastric tissue polymerase chain reaction (PCR), rapid urease test and biopsy histology) and non-invasive (serology, stool antigen test and urea breath test) [5]. The stool antigen tests consist of a qualitative technique based on enzyme-linked immunoassay (ELISA) or lateral flow assay that detects the presence of *H. pylori* antigen [1]. In addition, they have a sensitivity and specificity greater than 95% and they are reliable, simple, rapid and inexpensive [6]. The sensitivity and the specificity data vary between the different tests used. In recent meta-analyses, it was observed that these data correspond to a 91.4-92.4% in sensitivity and 91.9-93.0% in specificity [7]. Therefore, they constitute a good tool for the diagnosis of this infection, especially in the Primary Care area [1].

However, the stool antigen test can be falsely negative if there are a low density of *H. pylori* in the stomach and a low antigen load in the stool, which may be caused by the use of bismuth, proton pump inhibitor or antimicrobials, unformed or watery stool samples, and the time interval after eradication. In addition, the temperature and the interval between stool sample collection and measurement also affect the stool antigen test results [7].

In summary, an early diagnosis of this infection is essential in order to establish an effective treatment in patients and thus avoid the complications mentioned above. Consequently, the aim of this study was to compare the sensitivity and the specificity for the detection of *H. pylori* in human fecal samples, using *STANDARD *^*TM*^* F H. pylori Ag FIA* (SD Biosensor) on the *STANDARD* ^*TM*^* F2400* analyzer or the *LIAISON® Meridian H. pylori SA* (DiaSorin) on the *LIAISON® XL* platform, which was considered the reference standard.

## Material and Methods

A total of 133 human fecal samples from different patients suspected of *Helicobacter pylori* infection have been prospectively analyzed during three non-consecutive days and studied in parallel with *STANDARD* ^*TM*^* F H. pylori Ag FIA* (SD Biosensor Inc, Suwon, South Korea) and *LIAISON® Meridian H. pylori SA* (DiaSorin Inc, Minneapolis, Minnesota USA) assays. For the analysis of the results, the* LIAISON® Meridian H. pylori SA* has been taken as the reference assay, and the results obtained with this assay have been considered the true results. 48 samples were positive and 85 were negative for *LIAISON® Meridian H. pylori SA.*

The samples were processed according to the instructions provided by each company. Briefly, a sufficient amount of the stool samples was collected using a swab. The content of the swab was mixed with the commercial dilution buffer found in the test. Three drops of the mixture were then added at 30-second intervals onto the immunochromatography strip. This was introduced into the *STANDARD* ^*TM*^* F2400* analyser, reading the obtained results after 10 minutes. The samples were evaluated in parallel by *LIAISON® Meridian H. pylori SA*. In the cases where there was a discordant result between the two antigen detection tests, the *Allplex™ H. pylori/ClariR PCR* (Seegene Inc. Seoul, South Korea) was performed.

The statistical analysis was performed with Stata / IC 13.1 (StataCorp, Texas, USA). The 95% confidence interval (95% CI) values were calculated using the Wilson method. The degree of agreement between the reference technique (LIAISON) and the evaluated test was determined with Cohen's *Kappa* index. Positive predictive (PPV) and negative predictive values (NPV) were obtained according to the prevalence of *H. pylori* infection in our population.

## Results

The prevalence of positives in our population has been 23.2%, out of 11,596 samples analysed between 2018-2022.

The obtained results of positive or negative test of the LIAISON® and SD BIOSENSOR are shown in the Table 1.

**Table 1:** Results using SD BIOSENSOR and LIAISON® assays

		Diasorin LIAISON		
		Positive	Negative	Total
SD BIOSENSOR	Positive	44	5	49
	Negative	1	83	84
	Total	45	88	133

The sensitivity, specificity and positive or negative predictive value obtained in the *STANDARD™ F H. pylori Ag FIA* (SD Biosensor) test are shown on Table 2.

**Table 2:** Perfomance of the *STANDARD™ F H. pylori Ag FIA* (SD Biosensor) test

	Sensitivity (95% CI)	Specificity (95% CI)	PPV (95% CI)	NPV (95% CI)
*STANDARD™*	97.8%	94.3%	83.9%	99.3%
*F H. pylori Ag*	(88.2-99.9)	(87.2-98.1)	(68.9-92.4)	(95.3-99.9)
*FIA* (SD				
BIOSENSOR)				

In addition, these tests showed an agreement of 95.0% and a Cohen's *Kappa* index of 0.901 (almost perfect agreement).

We obtained a sensitivity of 97.8% (95% CI: 88.2-99.9), a specificity of 94.3% (95% CI: 87.2-98.1), a PPV of 83.9% (95% CI: 68.9-92.4) and a NPV of 99.3% (95% CI: 95.3-99.9).

Two negative samples of Diasorin LIAISON were positive in SD BIOSENSOR. PCR was performed to confirm these results, establishing that they were negative.

## Discussion

In this study, the sensitivity and specificity of the *STANDARD™ F H. pylori Ag FIA* (SD Biosensor) test for the determination of *H. pylori* infection were compared. The sensitivity was 97.8% while the specificity was 94.3%. These results are different from those seen in the studies of Yang, H., & Hu, B [7], which showed lower sensitivity and specificity. Also, the study by Benejat et al. [8] compared the RIDASCREEN® and RIDA®QUICK stool antigen tests for the diagnosis of *H. pylori* infection. The results showed that the first test had a sensitivity of 92.1% (95% CI: 79.2-97.3) and a specificity of 100% (95% CI: 96.1-100) while the second test had a sensitivity of 89.5% (95% CI: 75.9-95.8) and a specificity of 100% (95% CI: 96.1-100) [8]. Moreover, not all studies agree with a good result with similar immunochromatography assay tests, with 50% of sensitivity and 65% of specificity being seen in the study of Obaid, J. M. A. S. et al. [9]. Studies similar to ours using the Cohen *Kappa* index, showed a similarity of 0.885 [10].

The accuracy of monoclonal stool antigen test for the diagnosis of *H. pylori* infection was assessed in the systematic review and meta-analysis of Gisbert J.P. et al. [11]. The results of their study suited that monoclonal antigenic tests have a higher sensitivity than polyclonal tests, especially in post-treatment measure [11]. In our study, five false positives were obtained by the antigenic tests. The reasons for these failures could be various, such as temperature-related reasons [7, 10], watery stool samples [7], time lag between sample collection and measurement [7], poor storage of reagents [10] or heterogeneous distribution of antigens [10].

In addition, for invasive techniques, specific equipment and qualified personnel are needed to be able to take a good sample and perform the test well, whereas for non-invasive techniques, the procedure is simpler and faster. In the past, the specificity and sensitivity of non-invasive techniques could not compete with invasive techniques. However, improved PCR technology and antigenic tests are approaching the same results as invasive techniques. In the future, these techniques could replace biopsies for the detection of *H. pylori* infection [7].

These new and faster techniques could have a very similar diagnostic value to the invasive ones [7] or other non-invasive screening techniques [10]. Moreover, due to the high prevalence of *H. pylori* infection worldwide, this technique could show a high NPV, thus aiding to rule out infection.

## Conclusions

The diagnostic performance of *STANDARD™ F H. pylori Ag FIA* (SD Biosensor) has proved to  be an alternative to the chemiluminiscence antigenic test used in our population.

The *STANDARD™ F H. pylori Ag FIA* (SD Biosensor) on the *STANDARD™ F2400 *analyzer is a highly sensitive, specific and suitable assay for the detection of *H. pylori *in stool samples. The results have shown almost perfect concordance with the *LIAISON® H. pylori SA* assay. In addition, it also saves sample handling and working time as no sample pre-treatment is required.

## Data Availability

All data generated or analyzed during this study are included in this manuscript.
